# AI-based prostate volume estimation from multi-planar MRI under variable acquisition protocols

**DOI:** 10.1016/j.ejro.2026.100738

**Published:** 2026-02-25

**Authors:** Yao Lu, Tim Nikolaas Lindeijer, Tord Martin Ytredal, Andreas Bremset Alvestad, Alvaro Fernandez-Quilez

**Affiliations:** a Department of Computer Science and Electrical Engineering, University of Stavanger, Stavanger, Norway; b Department of Computer Science, University of Putra Malaysia, Malaysia; c SMIL, Department of Radiology, Stavanger University Hospital, Stavanger, Norway

**Keywords:** Magnetic resonance imaging, deep learning, knowledge-based, multi-planar, Abbreviated protocols

## Abstract

**Background:**

Prostate MRI protocols vary across institutions, with abbreviated protocols increasingly limited to axial plane acquisitions. Conventional deep learning (DL) models for prostate volume (PV) estimation typically require complete availability of annotated full imaging protocols during training and inference, limiting their adaptability in real-world clinical workflows. This study aimed to develop and evaluate a knowledge-based (KB) DL segmentation model that adapts to variable MRI acquisition protocols, including axial-only abbreviated protocols.

**Methods:**

This retrospective study included 629 multiparametric 3-Tesla prostate MRI exams (66.60 ± 7.50 years) from biopsy-confirmed patients. Manual segmentations by expert radiologists and ellipsoid-derived volumes per PI-RADS 2.1 (${\mathrm{PV}}_{\mathrm{ref}}$) served as reference standards. A 2D nnU-Net–based DL model with a KB contrastive loss was trained using only axial segmentations while incorporating unannotated orthogonal views (${\mathrm{PV}}_{\mathrm{KB}}$). Performance was compared to a fully supervised nnU-Net-based DL model trained with full multi-planar annotations and data (${\mathrm{PV}}_{\mathrm{DL}})$. Evaluation metrics included Dice Score Coefficient (DSC), Relative Volume Difference (RVD), Bland-Altman analysis, and intraclass correlation coefficient (ICC). Experiments simulated both full and abbreviated protocols (axial-only). Wilcoxon signed-rank tests were used to evaluate statistical differences in performance across configurations. Statistical significance was set at p < 0.05.

**Results:**

With full multi-planar input, the KB model achieved a DSC of 0.91 ± 0.03 and RVD of 2.1 ± 6.4%, comparable to the fully supervised ${\mathrm{PV}}_{\mathrm{DL}}$model. Under axial-only conditions, the KB model maintained high performance (DSC:0.88 ± 0.04,RVD:3.4 ± 7.1%). PV agreement with ${\mathrm{PV}}_{\mathrm{ref}}$remained excellent across conditions (ICC>0.90).

**Conclusions:**

The proposed KB DL model enables accurate and flexible PV assessment under variable MRI protocols without requiring segmentation masks beyond the axial plane.

## Highlights

• The proposed knowledge-based contrastive loss allows to adapt to abbreviated MRI prostate protocols.
• The proposed model achieves high accuracy in prostate volume estimation, even under axial-only abbreviated protocols.
• Agreement with ellipsoid-based reference volumes was high even across heterogeneous input conditions.

## 1 Background

Prostate cancer (PC) remains one of the most commonly diagnosed cancers among men worldwide [1]. In men with elevated prostate-specific antigen (PSA) levels, magnetic resonance imaging (MRI) plays an important role in detection, staging and management of the disease [2], [3]. T2-weighted (T2w) MRI provides high-resolution anatomical information of the prostate, supporting volumetric evaluations. However, its clinical use frequently depends on manual contouring by radiologists, a process that is time-intensive and shows variability even among experienced radiologists [4], [5], [6], [7].

To improve standardization and diagnostic consistency of MRI in PC, the Prostate Imaging Reporting and Data System (PI-RADS) was introduced in 2012 [7]. The current PI-RADS 2.1 guidelines recommend acquiring axial T2w images along with at least one orthogonal plane, either sagittal or coronal [7], [8], [9]. Prostate volume (PV) estimation is commonly performed using the ellipsoid formula (EF), which requires manual measurements of prostate height, width and depth across the multi-planar acquisition [5], [6]. While multi-planar acquisitions can support better anatomical characterization and more accurate PV estimation of the prostate, imaging protocols considerably vary across institutions [10]. Factors such as scanner availability, examination time constraints, and clinical priorities contribute to heterogeneity in the number and types of acquired planes [10], [11].

In recent years interest in and adoption of abbreviated MRI protocols has increased [8], [11]. This has further accentuated acquisition variability in PC imaging, where the protocol might be further adapted based on clinical needs [11]. As a result, the number of acquired T2w orthogonal views might be reduced, and in some cases, limited to the axial plane [7]. This can influence both the spatial resolution and anatomical coverage of the prostate, posing challenges for consistent prostate segmentation and PV estimation [12], [13]. The increasing variability in clinical workflows presents a paradigm shift where tools for PV estimation are required to reliably estimate PV under varying imaging conditions, including incomplete or reduced-plane protocols [4].

Deep learning (DL) methods have shown promise in automated prostate segmentation and PV estimation, offering a scalable alternative to reduce reliance on manual delineation [12], [14]. Architectures based on the U-Net model, particularly those developed using the nnU-Net framework and trained with axial T2w prostate MRI, have been widely adopted in PC research settings [13], [15]. However, models trained exclusively on axial MRI are often sensitive to acquisition variability [12], [13], [16]. To improve generalizability, multi-planar approaches incorporating axial, sagittal and coronal views together with corresponding manual segmentations masks for each view during training have been proposed [12], [13], [17]. While these approaches improve segmentation accuracy in controlled, in-silico experiments, they assume full multi-planar availability -including both imaging and annotations- during the training and/or use of the model [12], [13], [17]. This condition stands in contrast to the reality of clinical scenarios, where abbreviated protocols might be leveraged and view availability is often heterogeneous [11], [18].

To support clinical translatability, DL models for prostate segmentation and PV estimation should be designed to adapt to variable multi-planar T2w acquisitions. Informed DL is a class of approaches that aim to incorporate prior domain knowledge, such as physical properties or anatomical consistency, into the learning process of the DL algorithm [19]. Among these, knowledge-based (KB) loss functions offer a structured way to embed domain-relevant constraints such as a known geometry of the region of interest, simulate radiological reasoning, and introduce robustness in cases with incomplete or variable data availability [19], [20]. Although KB loss functions have been explored in other areas of medical imaging [19], [20], their application in the context of prostate segmentation and PV estimation remains largely unexplored.

In this work, we investigate the integration of a KB loss inspired by radiological practices into a DL-based segmentation framework and its effect on PV assessment in the presence of variable MRI acquisition protocols. We hypothesize that the proposed method will match or surpass PV estimation and prostate segmentation performance of a standard model trained and deployed with full multi-planar information, while maintaining comparable performance when only the axial view is available.

## 2 Methods

This retrospective multicenter study was conducted using data approved by the institutional or regional review board of all contributing centers. The requirement for informed consent was waived due to the retrospective, scientific, and de-identified use of data [21], [22].

### 2.1 Datasets

The study utilized data from two sources: the publicly available Prostate Imaging: Cancer Artificial Intelligence (PI-CAI) dataset [21] and an internal institutional dataset [22].

The PI-CAI dataset comprises multi-parametric MRI (mp-MRI) examinations collected retrospectively across four centers using scanners from two different vendors. MRI exams were acquired between January 2012 and December 2021 and included patients referred for imaging due to clinical suspicion of PC, based on elevated PSA levels or abnormal digital rectal examination findings. Patients with prior biopsy-confirmed PC or a history of treatment were excluded. The dataset consists of 1500 mp-MRI exams, containing multi-planar T2w and diffusion-weighted imaging. When available from clinical records, PSA levels (ng/ml), PV (ml), PSA density (ng/ml^2^), Gleason score and patient age (years) were included [21].

We also used a local institutional dataset [22], comprising 46 retrospectively collected mp-MRI exams acquired between January 2016 and December 2019. These exams were similarly obtained from patients under suspicion of PC, based on elevated PSA levels. The imaging protocol included multi-planar T2w and DWI sequences. Gleason scores, patient age and PV were included when available from clinical records. In both datasets, reported PVs were calculated using the ellipsoid formula in accordance with PI-RADS 2.1 guidelines [4], [23].

### 2.2 Study cohorts and ground truth segmentations

From the PI-CAI dataset, axial-plane segmentations of the prostate whole gland (WG) were available for 1001 mp-MRI exams at the time of the study. Of these, 204 segmentations originated from the publicly available ProstateX subset and were manually delineated by two radiology residents and two-board certified radiologists [24]. The remaining 797 segmentations were generated using a previously validated DL algorithm, under the supervision of one radiologist with over 7 years of experience [3], [21]

For the local institutional dataset, WG segmentations in the axial plane were available for all 46 exams. These were manually annotated by a radiology resident at the time of acquisition (A.B.A). All segmentations involving human supervision were performed using the open-source software ITK-SNAP v3.80 software (http://www.itksnap.org/).

**Fig. 1:**
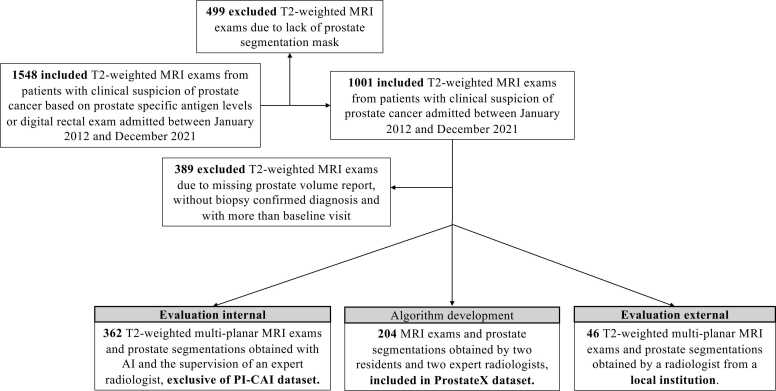
Patient inclusion criteria and resulting MRI exams used for algorithm development and for prostate volume evaluation.

A total of 408 patients were included in the final study cohort, comprising 362 exams from the PI-CAI dataset and 46 from the local dataset. Inclusion criteria were: (1) available T2w axial-plane WG segmentation (2) available T2w multi-planar information (3) available PV (4) no overlap with the 204 ProstateX patients used for the development of the prostate segmentation DL models [24] and (5) biopsy-confirmed diagnosis of PC. An overview of the dataset selection process is shown in Fig. 1.

### 2.3 MRI pre-processing

We used multi-planar T2w MRI acquisitions in combination with available axial WG segmentations. The PI-CAI dataset included images acquired using various commercial 1.5 Tesla (T) and 3.0 T scanners (Siemens Healthineers, Erlangen, Germany; Philips Medical Systems, Eindhoven, Netherlands) with a surface coil [21]. Axial in-plane resolution varied across exams (median: 0.5 mm; range: 0.2–0.8 mm), as did slice thickness (median: 3.5 mm; range: 2.2–5.0 mm). Sagittal and coronal showed similar acquisition variability with in—plane resolution (median: 0.5 mm; range: 0.2–0.8 mm) and slice thickness (median: 3.5 mm; range: 2.2–5.0 mm) comparable to axial images.

MRI data from our local institution were acquired using a 3.0 T Philips scanner (Philips Medical Systems, Eindhoven, Netherlands) with a surface coil [22]. Axial T2w images had an in-plane resolution of 0.5 mm× 0.5 mm and a slice thickness of 3.0 mm, whilst sagittal and coronal had an in-plane resolution of 0.34 mm× 0.34 mm and a slice thickness of 1 mm.

To reduce visual differences arising from scanner and acquisition heterogeneity, we applied pixel intensity normalization [25] and resampled all T2w images to a common reference space of 0.5 mm× 0.5 mm× 3.0 mm following PI-RADS 2.1 recommendations [23]. Image resampling used third-order spline interpolation [15]. For corresponding prostate WG segmentations, linear interpolation was applied [15].

### 2.4 Multi-planar DL-based prostate segmentation

**Fig. 2:**
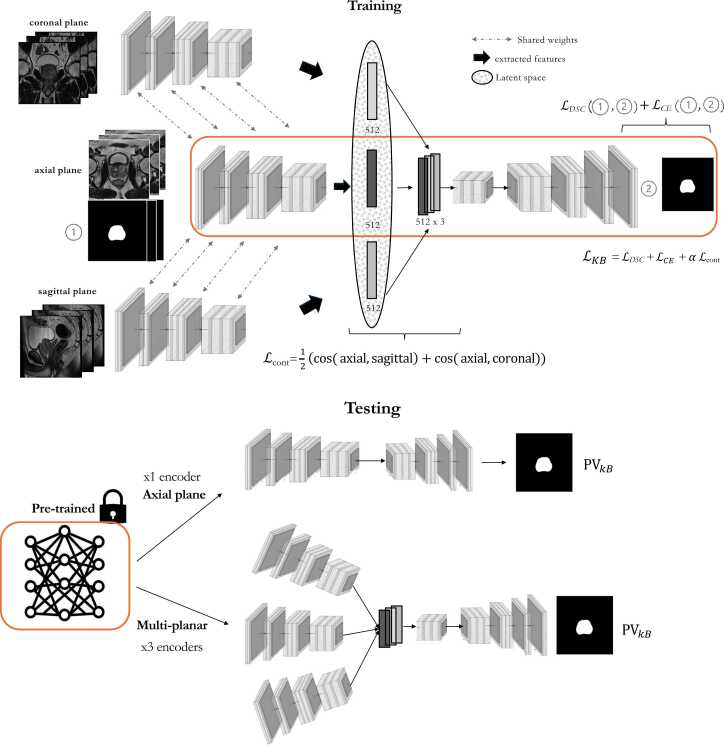
Overview of the proposed knowledge-based deep learning segmentation framework**. (a)** Training phase: A modified 2D nnU-Net architecture is used with three encoders, each processing a different T2-weighted plane (axial, sagittal, coronal). All encoders share weights. A knowledge-based loss ($L_{\mathrm{KB}})$ composed of the default nnU-Net loss (modified dice loss) with an additional similarity term based on cosine distance is applied to promote inter-view consistency. **(b)** Testing phase: The model adapts to available planes by activating only the necessary pre-trained encoders (e.g., axial-only or axial plus two orthogonal views). Prostate volume estimates from the knowledge-based model (${\mathrm{PV}}_{\mathrm{KB}})$ are compared with those from a standard nnU-Net without the contrastive loss (${\mathrm{PV}}_{\mathrm{DL}}$) and with reference volumes (${\mathrm{PV}}_{\mathrm{ref}})$ calculated by a radiologist using the ellipsoid formula as defined in PI-RADS 2.1.

We developed a customized 2D architecture based on the self-configuring nnU-Net framework, modified to incorporate multi-planar MRI inputs [13], [15], [26]. The model was designed to accommodate variable plane availability at deployment, including axial-only input scenarios commonly encountered in abbreviated protocols. As depicted in Fig. 2a, training supervision relied exclusively on axial segmentations. The choice of framework was based on its demonstrated performance in prostate segmentation tasks; additional technical details are available in prior publications [4], [15].

The architecture includes three parallel encoder branches that independently process axial, sagittal and coronal T2w images [17], [26] (Fig. 2a). Each encoder shares weights and follows the default 2D nnU-Net encoder structure. Only the axial encoder is connected to the decoder through skip connections, enabling direct propagation of high-resolution spatial features during training and deployment. The sagittal and coronal encoders are not connected to the decoder but contribute during training by informing a shared latent representation space (Fig. 2a). Unlike existing approaches, this design leverages complementary anatomical context from multiple planes without requiring non-axial segmentations during training.

Features from the three encoders are combined through a bottleneck module, which projects their outputs into a shared latent space of fixed dimensionality [15]. During deployment, the architecture can be flexibility adapted to the number of availability planes using the corresponding as needed and without altering the decoder or retraining the model (Fig. 2b).

The model was developed using 204 patients (median age: 66 years range: 48–83 years) multi-planar T2w MRI exams and corresponding prostate axial WG segmentations from the ProstateX subset [4], [24]. This development cohort was used solely for model development and excluded from evaluation (Fig. 1). A fivefold cross-validation strategy yielded five independently trained nnU-Net models. At inference, predictions were averaged to generate the final segmentation used for PV estimation.All models were trained for 300 epochs using TensorFlow (version 2.9.17), Python (version 3.9.12; Python Software Foundation, Wilmington, DE, USA) and using a single NVIDIA A100 GPU with 40 GB RAM (NVIDIA corporation, Santa Clara, California, USA).

### 2.5 Knowledge-based loss: orthogonal views consistency through contrastive learning

In prostate MRI, the axial view is considered the canonical plane for evaluating prostate anatomy and performing segmentation. Sagittal and coronal planes, in contrast, typically provide complementary spatial context often helping radiologists refine boundary interpretation [13], [17]. These observations suggest that although these views differ in appearance, corresponding slices across views depict the same underlying anatomy and are therefore expected to carry semantically similar information.

Based on this rationale, we introduced a KB-loss that encourages the DL segmentation model to learn consistent representations across views by exploiting the semantic similarity across views. Specifically, the model is guided to treat axial views as the central information source while aligning sagittal and coronal representations as complementary perspectives for prostate segmentation [27].

Consistency is enforced through a latent-space contrastive loss applied across the encoder branches corresponding to each view. During training, the sagittal and coronal encoders are encouraged to produce feature representations that closely align with those of the axial encoder for corresponding prostate anatomical locations (Fig. 2a). Alignment is quantified using a contrastive loss based on cosine similarity [8], [27]. The loss is applied between the axial and sagittal encoders, and between the axial and coronal encoders (Fig. 2a). This design reflects the canonical role of the axial plane in prostate volumetric evaluations, where orthogonal views as used as supportive sources of information. In doing so, the model is expected to learn that orthogonal views represent the same underlying anatomy and contribute to more robust learning, even in cases where only axial plane is available during its use (Fig. 2b).

The final KB-loss combines the default nnU-Net segmentation loss, including a dice and cross-entropy components [15] with a weighted contrastive loss to promote cross-view consistency. Whilst the default loss encourages voxel-level or pixel-level overlap between predicted and reference segmentations, the contrastive loss helps the model learn anatomical correspondence and consistency across views. Fig. 2a illustrates how this inter-view consistency is implemented, and the resulting KB loss.

### 2.6 DL-based prostate segmentation and volume assessment

To evaluate the effectiveness of our proposed KB-loss, we assessed its performance in prostate segmentation and PV estimation $\left({\mathrm{PV}}_{\mathrm{KB}}\right)$ under two varying multi-planar configurations: (1) multi-planar input (axial, coronal and sagittal) and (2) axial-only input. Performance was evaluated against two reference standards. First, we compared against manual annotations provided by experienced radiologists and with PV estimates derived with the EF (${\mathrm{PV}}_{\mathrm{ref}}$) suggested in PI-RADS v2.1. Following, performance was compared against a reference model trained in a fully supervised manner using segmentation masks for all three planes and assuming full multi-planar availability at deployment [17], [24]. This reference model performance in segmentation accuracy and PV estimation (${\mathrm{PV}}_{\mathrm{DL}}$) served as an upper performance bound under ideal training and deployment conditions.

Both the KB-loss model and the reference model share the same architecture (Fig. 2a), were trained and evaluated using identical data splits (Fig. 1), and employed the same ensemble strategy to generate final predictions. For the KB-loss model, the contrastive loss weight was set to 0.25 based on empirical tuning.

Segmentation quality was assessed quantitively using the Dice Score Coefficient (DSC) and the Average Surface Distance (ASD), two established metrics in prostate MRI studies [4], [24]. PV estimates were derived from the model’s segmentation masks by computing the total volume enclosed within the predicted prostate contours. To quantify agreement, we used the Relative Volume Difference (RVD) which expresses the deviation of the predicted volume from the reference volume. RVD values closer to zero indicate better alignment with the standard, with negative values reflecting underestimation and positive values indicating overestimation [4], [13], [28].

### 2.7 Statistical analysis

Continuous variables were reported as mean ± standard deviation (SD) for normally distributed variables and as a median interquartile range [Q1-Q3] when not normally distributed. Categorical variables were expressed as absolute counts and percentages (N(%)).

Comparisons between segmentation quality metrics (DSC, ASD), volumetric error (RVD) and PV estimates were performed between the KB-loss model and the fully supervised model. Statistical significance was evaluated using paired Student’s *t*-tests or Wilcoxon signed-rank tests, depending on data distributions.

Agreement between estimated volumes (${\mathrm{PV}}_{\mathrm{KB}}$ and ${\mathrm{PV}}_{\mathrm{DL}}$) and the reference ellipsoid-based volume (${\mathrm{PV}}_{\mathrm{ref}}$) was assessed using the intraclass correlation coefficient (ICC). ICC values were interpreted as follows: < 0.50 = poor, 0.50–0.75 = moderate, 0.75–0.90 = good and > 0.90 = excellent. Bland-Altmann plots were used to evaluate bias and limits of agreement between predicted and reference volumes. Comparisons were first conducted between ${\mathrm{PV}}_{\mathrm{DL}}$ and ${\mathrm{PV}}_{\mathrm{ref}}$, and then between ${\mathrm{PV}}_{\mathrm{KB}}$ and ${\mathrm{PV}}_{\mathrm{ref}}$. Volume estimation distributions of ${\mathrm{PV}}_{\mathrm{DL}},{\mathrm{PV}}_{\mathrm{KB}}$ and ${\mathrm{PV}}_{\mathrm{ref}}$ were visualized using boxplots All comparisons were conducted across the two different plane availability scenarios.

All statistical analyses were conducted using Python 3.9 (https://www.python.org/) and the statsmodels 0.14.0 library (https://www.statsmodels.org/stable/index.html). A two-sided p-value < 0.05 was considered statistically significant.

## 3 Results

The internal evaluation cohort included 362 patients (mean age: 66.47 ± 7.49 years; mean prostate volume: 59.30 ± 32.43 ml), while the external evaluation cohort consisted of 46 patients (mean age: 65.56 ± 7.70 years; mean prostate volume: 95.74± 11.43 ml). Across both datasets, clinical profiles reflected a wide range of prostate sizes and Gleason scores. Table 1a and Table 1b depicts the demographic and clinical characteristics of the internal and external cohorts, respectively.

**Table 1:** Demographic and clinical characteristics of (a) development and internal evaluation study participants and (b) external evaluation study participants.

**a**	
***Characteristic***	***Patients*** *(N = 362)*
Age (years)	66.47 ± 7.49
PSA (ng/ml)	8.70 (6.00 and 12.45)
Prostate volume (ml)	59.30 ± 32.43
≤*35*	82 (22.65%)
>*35 and*<*50*	81 (22.38%)
≥*50*	199 (54.97%)
PSAd (ng/ml^2^)	0.17 (0.11 and 0.25)
Biopsy Type	
*Systematic*	116 (32.04%)
*MRI guided*	136 (37.58%)
*MRI (+Systematic)*	100 (27.62%)
*Radical prostatectomy*	10 (2.76%)
Biopsy results	
*Negative*	135 (37.29%)
*Gleason score*<*7*	87 (24.04%)
*Gleason score*≥*7*	140 (38.67%)
**b**	
Age (years)	65.56 ± 7.70
PSA (ng/ml)	N/A
Prostate volume (ml)	95.74± 11.43
≤*35*	0 (0.00%)
>*35 and*<*50*	0 (0.00%)
≥*50*	46 (100.00%)
PSAd (ng/ml^2^)	N/A
Biopsy Type	
*Systematic*	0 (0.00%)
*MRI guided*	44 (95.65%)
*MRI (+Systematic)*	0 (0.00%)
*Radical prostatectomy*	2 (4.35%)
Biopsy results	
*Negative*	13 (28.26%)
*Gleason score*<*7*	16 (34.78%)
*Gleason score*≥*7*	17 (36.96%)

### 3.1 Prostate segmentation and volume estimation performance

As shown quantitatively in Table 2, in the internal evaluation cohort the KB model with multi-planar input achieved a significantly higher segmentation performance (88.51 ± 9.48%; ASD: 0.54 mm [0.33–1.72]) and lowest volume deviation from the ellipsoid-estimated volume reference ${\mathrm{PV}}_{\mathrm{ref}}$ (RVD: −8.03± 11.68%).

**Table 2:** Effect of knowledge-based architecture in the presence of axial plane and complete multi-planar information in deep learning-based prostate volume assessment and prostate segmentation in the internal evaluation cohort.

	***PV and segmentation assessment*** *(N = 362)*			***p value***	
***Value***	*DL algorithm* *KB (axial)*^*2*^	*DL algorithm* *(multi-planar)*^*1*^	*DL algorithm* *KB (multi-planar)*^*3*^	*1,2*	*1,3*
DSC (%)	78.00 ± 11.41	80.66 ± 9.00	88.51 ± 9.48	**< 0.001**^✝^	**< 0.001**^✝^
ASD (mm)	0.76 (0.54 and 5.88)	0.82 (0.58 and 6.15)	0.54 (0.33 and 1.72)	**< 0.001**^✝^	**< 0.001**^✝^
**RVD (%)**	-11.89 ± 12.62	-11.53 ± 16.68	-8.03± 11.68	0.569	**< 0.001**^✝^
PV (ml)	65.59 ± 34.50	65.37 ± 35.03	63.61 ± 34.18	0.683	**< 0.001**^✝^

^✝^*p*^-^values < 0.05 were considered statistically significant.

DL = Deep learning; KB = Knowledge-based; DSC = Dice score coefficient.

ASD = Average surface distance; RVD = Relative volume difference; PV = Prostate volume.

**Fig. 3:**
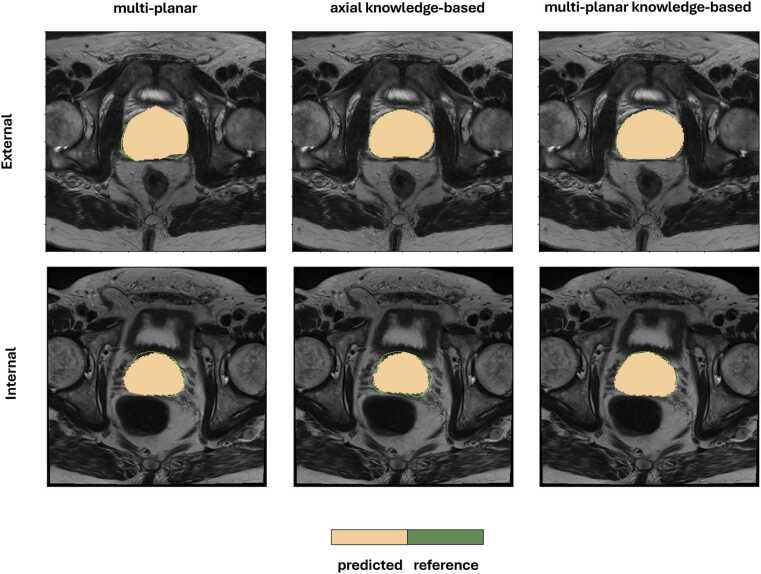
Qualitative comparison of prostate segmentations across models and settings. Slices from two T2w MRI exams, one from the external test set (top row) and one from the internal test set (bottom row). Left column shows prostate contours predicted by the baseline multi-planar deep learning model without knowledge-based training. Middle column shows predictions from the knowledge-based model using only axial plane input. Right column shows predictions from the full multi-planar knowledge-based model. Ground truth reference contours are shown in green in all images, whilst predictions are depicted in light orange.

When in the presence of abbreviated protocols with only axial plane availability the KB model preserved the segmentation and volume estimation performance (DSC: 78.00 ± 11.41%; RVD: −11.89±12.62%) when compared to the reference model trained and tested with full availability of multi-planar data (DSC: 78.00 ± 11.41%; RVD: −11.89±12.62%) No statistically significant differences were found between these two in RVD (p = 0.569) or PV estimation (p = 0.683). Visual examples of segmentation outputs from the baseline and KB models under multi-planar and axial-only settings are presented in Fig. 3. Qualitatively, the KB model demonstrated improved delineation especially in regions with ambiguous boundaries.

As shown in Table 3, the KB model significantly outperformed in the external cohort the reference model in segmentation accuracy (DSC: 91.64 ± 7.80% vs 77.26 ± 10.69%) and PV estimation (RVD: −4.98± 5.67% vs 4.99 ± 10.31%). When limited to axial input, the KB model remained consistent and showed better generalization abilities in segmentation accuracy when compared to the fully supervised multi-planar reference model (DSC: 78.76 ± 10.61% vs 77.26 ± 10.69%) and PV estimation (RVD: 4.99± 10.31% vs 5.61 ± 10.44%). Segmentation differences are presented qualitatively in Fig. 3.

**Table 3:** Effect of knowledge-based architecture in the presence of axial plane and complete multi-planar information in deep learning-based prostate volume assessment and prostate segmentation in the external evaluation cohort.

	***PV and segmentation assessment*** *(N = 46)*			***p value***	
***Value***	*DL algorithm* *KB (axial)*^*2*^	*DL algorithm* *(multi-planar)*^*1*^	*DL algorithm* *KB (multi-planar)*^*3*^	*1,2*	*1,3*
DSC (%)	78.76 ± 10.61	77.26 ± 10.69	91.64 ± 7.80	**0.02**^✝^	**< 0.001**^✝^
ASD (mm)	0.65 (0.34 and 24.13)	0.67 (0.35 and 24.94)	0.21 (0.03 and 21.24)	0.07	**< 0.001**^✝^
**RVD (%)**	4.99 ± 10.31	5.61 ± 10.44	-4.98± 5.67	0.10	**< 0.001**^✝^
PV (ml)	92.03 ± 22.85	91.50 ± 22.87	101.11 ± 19.10	0.11	**< 0.001**^✝^

^✝^*p*^-^values < 0.05 were considered statistically significant.

DL = Deep learning; KB = Knowledge-based; DSC = Dice score coefficient.

ASD = Average surface distance; RVD = Relative volume difference; PV = Prostate volume.

### 3.2 Agreement in prostate volume estimation

**Fig. 4:**
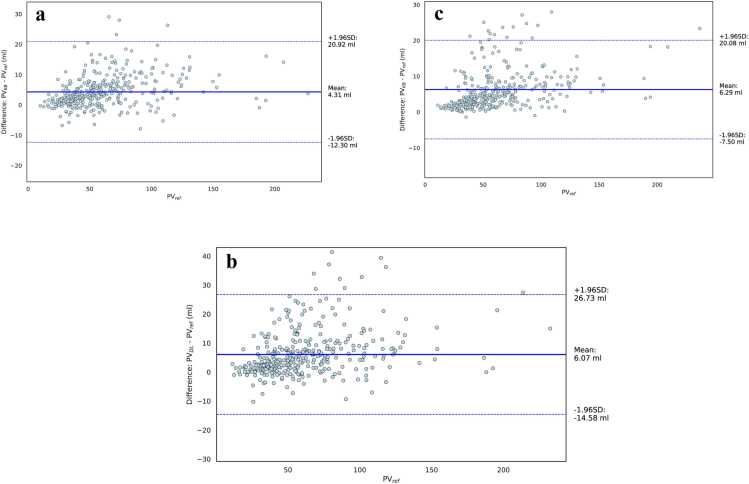
**(a)** Bland-Altman (B-A) plot comparing the prostate volume ${\mathrm{PV}}_{\mathrm{KB}}$ obtained with axial plane and the knowledge-based (KB) architecture with the volume resulting from applying the ellipsoid formula ${\mathrm{PV}}_{\mathrm{ref}}$. (**b**) B-A plot comparing ${\mathrm{PV}}_{\mathrm{DL}}$ calculation resulting from the deep learning algorithm without KB loss and axial, sagittal and coronal planes with the volume resulting from applying the ellipsoid formula ${\mathrm{PV}}_{\mathrm{ref}}$. (**c**) B-A plot comparing ${\mathrm{PV}}_{\mathrm{KB}}$ obtained with axial, sagittal and coronal plane and the KB architecture with the volume resulting from applying the ellipsoid formula ${\mathrm{PV}}_{\mathrm{ref}}$. In all instances, the solid lines represent the mean difference and the dashed lines the limits of the agreements, calculated as mean difference ± 1.96 SD.

**Fig. 5:**
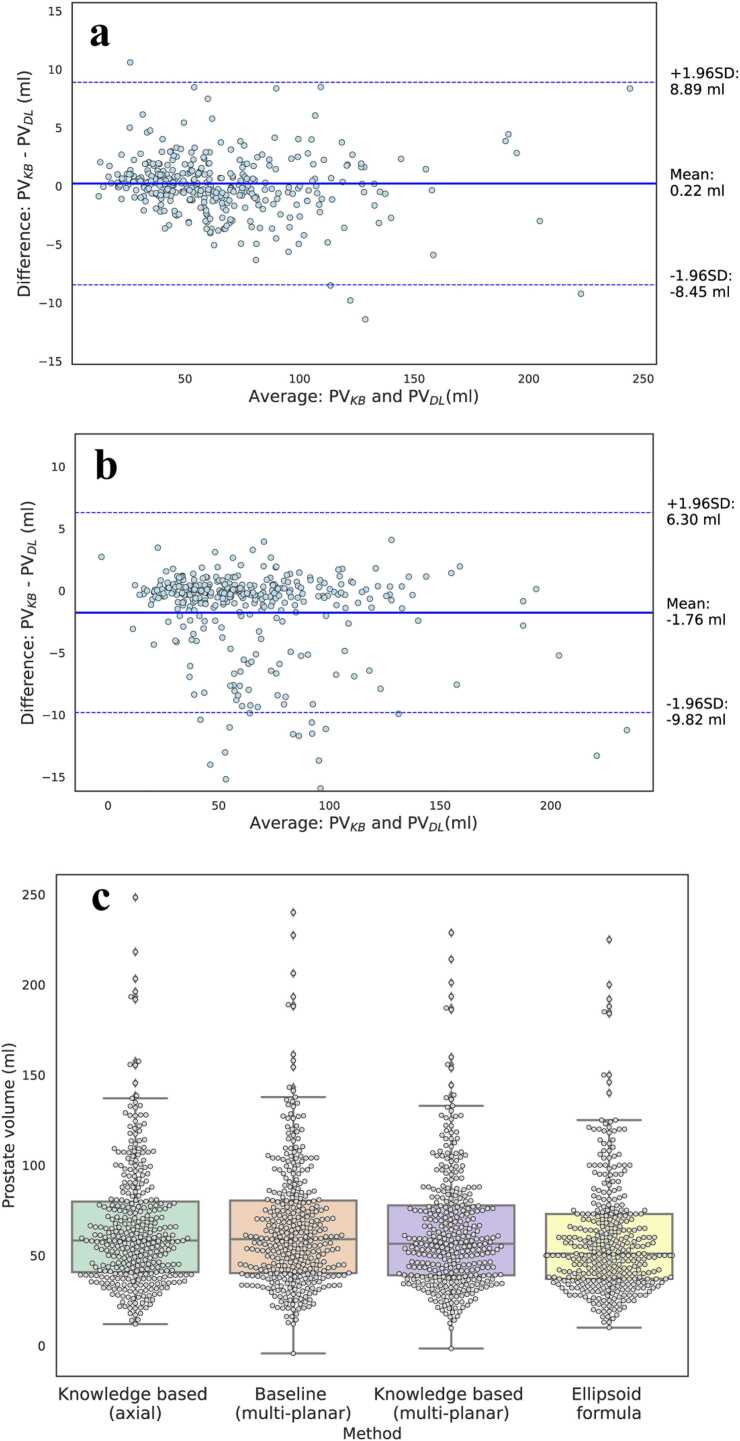
Bland-Altman plot comparing prostate volumes measured by the multi-planar deep learning algorithm ${\mathrm{PV}}_{\mathrm{DL}}$ and multi-planar KB architecture ${\mathrm{PV}}_{\mathrm{KB}}$ in **(a)** an axial-only and **(b)** multi-planar setting. The solid lines represent the mean difference and the dashed lines the limits of the agreements, calculated as mean difference ± 1.96 SD. **(c)** Boxplot depicting the distribution of calculated prostate volumes by the multi-planar deep learning algorithm, knowledge-based architecture and ellipsoid formula, respectively.

**Fig. 6:**
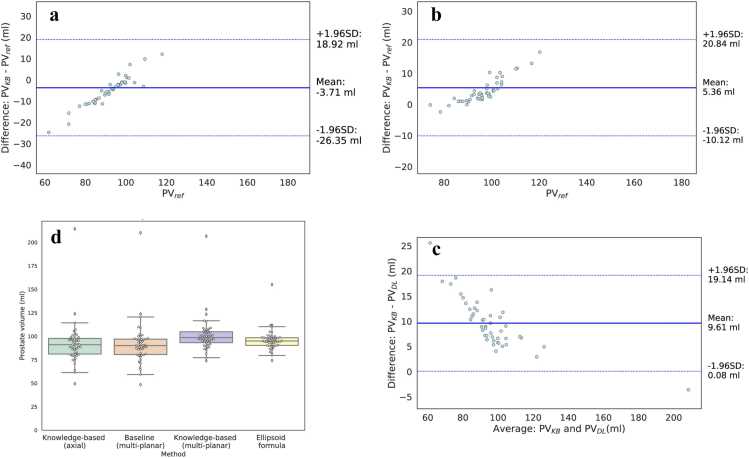
**(a)** Bland-Altman plot comparing prostate volumes in the external evaluation dataset obtained with axial plane and the knowledge-based (KB) architecture against the PI-RADS 2.1 ellipsoid formula ${\mathrm{PV}}_{\mathrm{ref}}$, **(b)** multi-planar KB architecture ${\mathrm{PV}}_{\mathrm{KB}}$ against the PI-RADS 2.1 ellipsoid formula ${\mathrm{PV}}_{\mathrm{ref}}$ and **(c)** deep learning algorithm ${\mathrm{PV}}_{\mathrm{DL}}$ and multi-planar KB architecture ${\mathrm{PV}}_{\mathrm{KB}}$. The solid lines represent the mean difference and the dashed lines the limits of the agreements, calculated as mean difference ± 1.96 SD. **(d)** Boxplot depicting the distribution of calculated prostate volumes by the multi-planar deep learning algorithm, knowledge-based architecture and ellipsoid formula, respectively.

Analyses of the agreement comparing ${\mathrm{PV}}_{\mathrm{KB}}$ and ${\mathrm{PV}}_{\mathrm{DL}}$ against ${\mathrm{PV}}_{\mathrm{ref}}$ as the reference standard are presented as a Bland-Altman plot in Fig. 4 and Fig. 5 for the internal cohort and Fig. 6 for the external cohort, respectively. In both cases, the mean difference and 95% limits of agreement were narrower for the KB multi-planar model compared to the multi-planar DL reference model. In the axial-only setting, the KB model maintained acceptable agreement with ${\mathrm{PV}}_{\mathrm{ref}}$ with a slightly wider confidence bounds (Fig. 5a and Fig. 6a) but no evidence of systematic over- or underestimation. The box plot in Fig. 5c and Fig. 6d visually depicts the distributions for all compared volumes in the internal and external dataset, respectively.

As depicted in Figure S1 and Figure S2, PV estimates from the KB model ${\mathrm{PV}}_{\mathrm{KB}}$ showed strong agreement with ${\mathrm{PV}}_{\mathrm{ref}}$. In the internal dataset, the KB model with axial input showed comparable agreement to the multi-planar baseline model in both correlation (0.90 vs 0.89) and visual concordance (Figure S1). Similar findings were observed in the external dataset (Figure S2). The ICC (mean [95% CI) was significantly larger for the KB model with multi-planar input (internal: 0.98; external: 0.90) and axial only input (internal: 0.98; external: 0.88) than the DL multi-planar reference (internal: 0.96; external: 0.87) in both internal and external datasets. In the internal dataset agreement was excellent with ${\mathrm{PV}}_{\mathrm{ref}}$, whilst in the external it was good.

## 4 Discussion

This retrospective study presents a KB-based DL segmentation model for PV assessment, designed to accommodate the increasing adoption of abbreviated MRI acquisition protocols seen in clinical practice. By introducing a contrastive loss that encourages semantic consistency across prostate orthogonal views without requiring segmentation masks beyond the axial plane during training, our approach leverages the canonical nature of the axial plane and supportive role of orthogonal views in radiological practice.

Our findings demonstrate several key outcomes. First, the proposed KB model significantly improves segmentation and PV estimation accuracy of a fully supervised reference model trained and deployed with complete multi-planar imaging and annotations. This was indicated by reduced RVD, higher DSC and stronger agreements in Bland-Altman plots and ICC. Second, when evaluated with axial-only planar information the KB model performance in prostate segmentation and PV estimation was comparable to the fully supervised multi-planar reference model. Third, the previous observations were consistent across both internal and external test sets highlighting the potential robustness of our approach. Collectively, these findings support the hypothesis that the KB model can match or improve PV estimation and prostate segmentation performance of a standard model trained and deployed with full multi-planar information, whilst maintaining comparable performance in the presence of axial-only abbreviated protocols.

Previous studies have proposed standard DL segmentation methods leveraging multi-planar information to improve performance in prostate MRI tasks [13], [17], [29]. Meyer et al. and Shanmugalingam et al. trained isotropic and anisotropic U-Net-based multi-planar segmentation models on cohorts from public and private datasets, achieving DSCs of 90–93% and absolute RVDs ranging from 1.12% to 23%. Whilst direct comparison is limited by dataset differences, our KB model shows performance within these reported ranges. Unlike previous studies, our method does not require annotations beyond the axial plane during training and retains flexibility during deployment when only partial multi-planar data is available. From a clinical perspective, this suggests that our method might be more suitable than others in real-world clinical workflows where abbreviated protocols are increasingly common [11].

While informed DL strategies have been explored in other domains [19], [20], efforts to explicitly integrate clinical radiological reasoning into model design are limited [19], [20], [30]. Our method operationalizes radiologist-like decision-making by embedding practice-motivated knowledge into the models’ learning objective. Specifically, that orthogonal planes are complementary yet non-redundant to axial plane information. By doing so, we promote a more interpretable learning process aligned with clinical reasoning and interpretation [31], [32]. Previous work has emphasized the importance of model transparency and clinician understanding in promoting effective human-AI collaboration [20], [32], [33]. Approaches rooted in human expertise and intuitive reasoning may be thus an important piece to building clinician trust and promotion adoption of AI tools in radiology practice [32].

Attempts to address systematic differences in PV calculation in scenarios with heterogenous data have shown improved efficacy at the cost of requiring significantly large data volumes or complex data acquisition schemes with low feasibility in clinical practice [29], [31], [33]. These may be difficult to scale and are misaligned with ongoing movements toward imaging sustainability, including abbreviated protocols [34]. In contrast, our method offers a principled mechanism for increasing robustness to MRI view availability while minimizing the need for additional training annotations or redundant acquisitions. This overall provides a practical proactive method towards sustainable clinical deployment without compromising performance nor robustness [34].

Our study has several limitations. First, as a retrospective analysis, prospective validation is warranted to confirm causal relationships. Second, the approach was implemented using a specific DL architecture and performance may vary across network types. Future exploration of more complex architectures might yield better results. Third, whilst reliance on ensemble architectures is common, the examination of their effect on the results could provide future valuable insights. Additionally, while external validation was included, broader testing across clinical characteristics, scanner types and acquisition protocols would strengthen generalizability of the findings. Finally, whilst we empirically show the effectiveness of the cosine similarity-based contrastive loss, future work may explore more sophisticated measures to further improve volume estimation performance.

## CRediT authorship contribution statement

**Yao Lu:** Writing – original draft, Methodology, Investigation, Formal analysis, Data curation. **Lindeijer Tim:** Writing – review & editing, Software, Methodology, Investigation. **Ytredal Tord:** Writing – review & editing, Software, Methodology, Investigation. **Alvaro Fernandez-Quilez:** Writing – review & editing, Writing – original draft, Supervision, Project administration, Funding acquisition, Conceptualization. **Alvestad Andreas:** Writing – review & editing, Validation, Supervision, Investigation.

## Ethics approval and consent to participate

The use of the data was approved by the institutional or regional review board of all the contributing centers of the study: Prostaat Centrum Noord-Nederland: (2018) IRB 2018–597; St Olav's Hospital, Trondheim University Hospital: (2017) REK 2017/576; Radboud University Medical Center: CMO (2016) 2016–3045; Ziekenhuisgroep Twente: (2023) ZGT 23–37 and Stavanver University Hospital: (2019) REK 2019/272 Informed consent was waived given the retrospective, scientific and de-identified use of the data.

## Authors' contributions

Y.L., T.N.L., T.M.Y: data curation, deep learning model development and evaluation. A.B.A.: Data acquisition A.F.Q.: Conceptualization, supervision, writing-review and funding acquisition. All authors read and approved the final manuscript.

## Relevance statement

A knowledge-based DL segmentation model allows consistent and accurate prostate volume estimation under full or abbreviated multi-planar MRI protocols, increasing adaptability to real-world clinical workflows.

## Funding

This study was supported by the 10.13039/100014513University of Stavanger. The funders had no role in the study design, data collection, data analysis, data interpretation, or writing of the report. The corresponding authors had full access to all the data in the study.

## Declaration of Competing Interest

The authors declare that they have no competing interests.

## Data Availability

The datasets used and/or analyzed during the current study are available from the corresponding author (E-mail: alvaro.f.quilez@uis.no) on reasonable request.
