# Supplementary material for: AI-based prostate volume estimation from multi-planar MRI under variable acquisition protocols
Source: Eur J Radiol Open. 2026 Feb 25;16:100738. doi: 10.1016/j.ejro.2026.100738 (PMC12954287; doi:10.1016/j.ejro.2026.100738)
Supplement: Supplementary file 1 — Supplementary material [file mmc1.docx]

**Supplementary Figures**

**b**

**a**

**c**

**Figure S1.** Spearman correlation analysis for the internal evaluation dataset **(a)** for the prostate volume measured by the PI-RADS 2.1 ellipsoid formula $\mathrm{PV}_{ref}$ and by the knowledge-based architecture with axial plane $\mathrm{PV}_{KB}$. **(b)** between the prostate volume from the PI-RADS 2.1 ellipsoid formula $\mathrm{PV}_{ref}$ and by the multi-planar deep learning algorithm $\mathrm{PV}_{DL}$.and **(c)** analysis for the prostate volume measured by the PI-RADS 2.1 ellipsoid formula $\mathrm{PV}_{ref}$ and by the multi-planar knowledge-based architecture $\mathrm{PV}_{KB}$. Highlighted regions correspond to the 95% confidence interval calculated through bootstrapping.

**Figure S2.** Spearman correlation analysis for the external evaluation dataset **(a)** for the prostate volume measured by the PI-RADS 2.1 ellipsoid formula $\mathrm{PV}_{ref}$ and by the knowledge-based architecture with axial plane $\mathrm{PV}_{KB}$. **(b)** for the prostate volume measured by the PI-RADS 2.1 ellipsoid formula $\mathrm{PV}_{ref}$ and by the multi-planar knowledge-based architecture $\mathrm{PV}_{KB}$. Highlighted regions correspond to the 95% confidence interval calculated through bootstrapping.
